# Born in Bradford’s Age of Wonder cohort: protocol for adolescent data collection

**DOI:** 10.12688/wellcomeopenres.20785.1

**Published:** 2024-02-12

**Authors:** Katy A. Shire, Alex Newsham, Atif Rahman, Dan Mason, David Ryan, Deborah A. Lawlor, Gail Opio-Te, Hannah Nutting, Jane West, John Pickavance, Josie Dickerson, Kate E. Pickett, Laura Lennon, Lydia Gunning, Mark Mon-Williams, Sean Smith, Simon Gilbody, Sufyan Dogra, Theresa Walsh, Rosemary McEachan, John Wright

**Affiliations:** 1 Bradford Institute for Health Research, Bradford Teaching Hospitals NHS Foundation Trust, Bradford, England, BD9 6RJ, UK; 2 Institute of Population Health, Department of Primary Care and Mental Health, University of Liverpool, Liverpool, England, L69 3GL, UK; 3 MRC Integrative Epidemiology Unit, University of Bristol, Bristol, England, BS82BN, UK; 4 Population Health Science, Bristol Medical School, University of Bristol, Bristol, England, BS8 2BN, UK; 5 Bristol NIHR Biomedical Research Centre, University Hospitals Bristol NHS Foundation Trust, University of Bristol, Bristol, England, BS8 2BN, UK; 6 Department of Health Sciences, University of York, York, England, YO10 5DD, UK; 7 School of Psychology, University of Leeds, Leeds, England, LS29JT, UK

**Keywords:** Born in Bradford, Adolescent, mental health, wellbeing, ethnicity, obesity, cognitive development, cohort

## Abstract

**Background:**

Adolescence and transition into adulthood are periods shaping life-long mental health, cardiometabolic risk, and inequalities. However, they are poorly studied and understood. By extending and expanding the Born in Bradford (BiB) cohort study through this period using innovative, co-produced approaches to collect and analyse data, we aim to understand better the interplay of factors that influence health and wellbeing, and inform/evaluate interventions to improve them and reduce inequalities.

**Protocol:**

BiB Age of Wonder (AoW) is a large, whole city cohort that will capture the contemporary lived experience amongst multi-ethnic adolescents progressing into young adulthood. We will collect repeated data from existing BiB participants and their peers (N~30,000 adolescents). The protocol for the first phase of the quantitative methods, involving survey measurements and health assessments in mainstream secondary schools is described here. We describe the co-production behind these methods, and lessons learned from the first year of data collection.

## Plain Language Summary

Born in Bradford have been following the health and wellbeing of over 13,000 Bradford children since they were born. This group of children are now in their teenage years – a time that is crucial for their future mental and physical health. Age of Wonder aims to capture this journey through adolescence and early adulthood with up to 30,000 young people in Bradford over 7 years. In the first phase of this project, data collection is taking part in secondary schools in Bradford. This protocol describes how this data collection is currently being carried out in the schools. Young people aged 12-15 are being asked to complete questionnaires, covering topics such as mental and physical health. These topics have been designed with groups of young people, schools and other partners, to make sure we are capturing data on the things most important to young people. Those in Year 9 (13-14 years old), are also asked to take part in physical health measurements such as height, weight, blood pressure and a blood sample, as well as computer-based assessments of cognition (memory), movement and language. There have been a number of lessons learned from the first full year of data collection, such as how to make the research as easy as possible for schools to take part in when they have so many competing demands on their time.

## Introduction

### Born in Bradford

Born in Bradford (BiB) is an internationally recognised birth cohort study situated within the youngest and one of the most ethnically and culturally diverse cities in the UK ( City of Bradford Metropolitan District Council, 2022). Between 2007 and 2011, 12,453 pregnant women were recruited during their routine glucose testing appointment at Bradford Royal Infirmary, resulting in an initial cohort of 13,776 children. Cord blood samples were taken at birth and parents provided detailed questionnaire data. Follow-up data has included physical health measures taken by primary school nurses ( West *et al.*, 2018), whole cohort record-linkage to health and educational data, detailed bespoke data collection with sub-samples ( Kelly *et al.*, 2017), and addition of biomarker data using stored biological samples, including genome-wide epigenetic data and metabolomic data ( Narasimhan *et al.*, 2016).

BiB’s most recent follow-up study focussed on children when they were aged 7–11 years old ( Bird *et al.*, 2019). Known as BiB Growing Up, this research explored social and emotional wellbeing, growth, adiposity and cardio-metabolic health, and cognitive and sensorimotor function, collecting data on over 9,000 BiB children and over 596,000 parents. When the children started primary school, BiB expanded the cohort to include an additional 10,201 of their peers and thus provide whole classroom assessments of cognitive development and well-being ( Hill *et al.*, 2021; Shire *et al.*, 2020). This approach was well received by both schools and parents and proved efficient and inclusive. As a result, we were able to provide schools with important information about children’s strengths and difficulties, helping them to identify children in need of additional support.

### Age of Wonder

The BiB children have now reached adolescence. This critical period involves rapid biological, psychological and social development, and is a high risk period for the onset of mental ill-health ( Blakemore, 2019; Paus *et al.*, 2008). Indeed, data collected on BiB children during primary school found that more than half of the 16,000 children who took part reported being bullied some or all of the time, a third said they kept their worries to themselves and a quarter worried all the time about how much money their families have ( Pickett *et al.*, 2021). All three are key experiences of socioeconomic contexts that are highly relevant to mental health across the life-course. In addition, the cohort is experiencing adolescence against the backdrop of two years of disrupted education, and further disruption to society at large as a result of the covid-19 pandemic.

BiB Age of Wonder (AoW) is a seven year research programme, encompassing a continuation of the intensive data collection with the BiB cohort during their adolescence and early adulthood. It also involves its expansion to include non-BiB children in the same year groups, who live or go to school in the Bradford district, resulting in a sample size of up to 30 000 young people. This will enhance the BiB resource so that researchers can undertake transformational research to understand young people’s mental and physical health and wellbeing, and develop solutions to address these problems, in a multi-ethnic socioeconomically diverse population that is experiencing an unprecedented time of global, societal, political, economic and environmental change.

### Aim

AoW aims to understand the factors that influence health and wellbeing between the ages of 12–21 years in a multi-ethnic city-wide cohort. This will inform and evaluate interventions to improve health and wellbeing and reduce inequalities.

### Objectives

1. To co-produce the Age of Wonder research programme with young people and stakeholders to ensure that it reflects the needs of young people (Work Package 1)
2. To explore determinants of health and wellbeing in young people between the ages of 12–21 using mixed quantitative (Work Package 2), longitudinal qualitative methods (Work Package 3) and ecological momentary assessment measures (Work Package 4)
3. To consent young people when they turn 16 to routine data linkage and for long term follow-up as part of the Born in Bradford research programme (Work Package 5)
4. To embed the Age of Wonder research programme into school curricula and work with young people and schools to inspire a new generation of researchers (Work Package 6)
5. To develop a platform for the co-production and evaluation of evidence based adolescent mental health interventions (delivered as a part of usual practice) by creating an innovative interventional arm to the Age of Wonder cohort (Work Package 7)
6. To develop methods of open access to Age of Wonder data including a biobank of up to 16,000 biological samples to maximize discoverability (Work Package 8).
7. To rapidly translate emerging findings into practice and integrate the Age of Wonder programme within the City’s health, education and voluntary sector communities as part of a City Collaboratory (Work Package 9)

**Figure 1.**
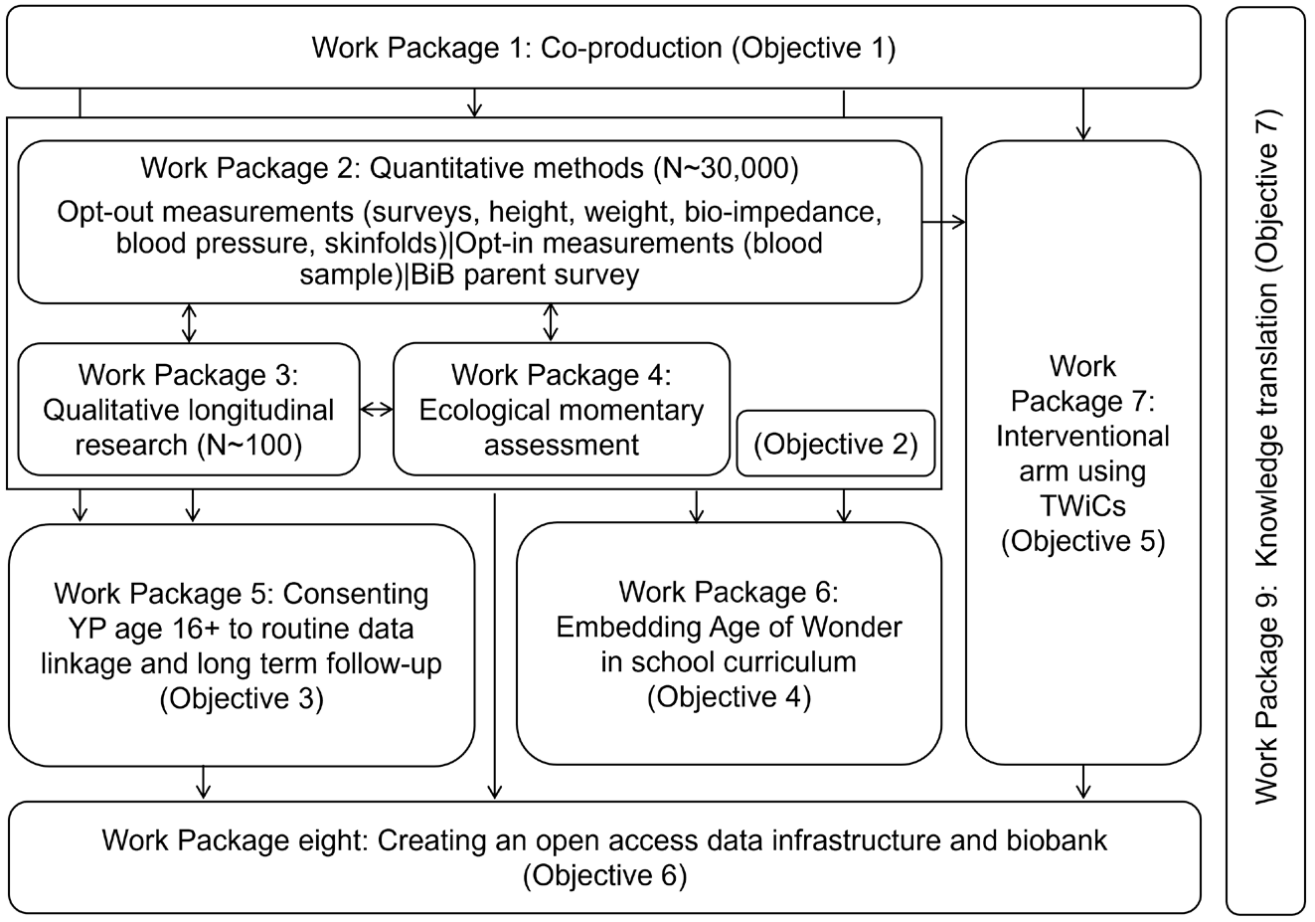
Overview of key Work Packages within Age of Wonder. Shows the interlinking work packages that make up the Born in Bradford (BiB) Age of Wonder (AoW) research programme, and the objectives each work package relates to. TWiCs = Trials Within Cohorts.

Figure 1 gives an overview of the Work Packages referenced. Co-production (Work Package 1) is the underpinning Work Package of the entire research programme. Work Packages 2–4 are linked mixed methods data collection Work Packages, with emerging findings being used to identify research priorities and methods throughout the course of the project. For example, the rich qualitative data on details of teenage stories gained through creative, artistic and qualitative longitudinal research methods ( Dogra *et al.*, 2023). Work Package 5 will focus on maximizing engagement with and ownership of the project with young people aged 16+ with a view to obtaining informed consent for their own participation and routine data linkage for long-term follow-up within the BiB project. Work Package 6 will work with young people and teachers to embed AoW within school curricula, with a view to raising aspiration and learning in research and Science, Technology, Engineering and Mathematics topics (STEM). Work Package 7 will run in parallel and will work with service providers and commissioners to co-produce evidence based interventions to improve mental health, the evaluation of which will be embedded in the AoW research platform, using novel evaluation methods such as trials within cohorts (TWiCs). Consent for TWiCs evaluations will be embedded in all consent activities in the cohort. Work Package 8 will create an open access data infrastructure platform and biobank to maximize visibility and use of the AoW platform. Finally Work Package 9 will focus on knowledge translation and dissemination activities.

**Figure 2.**
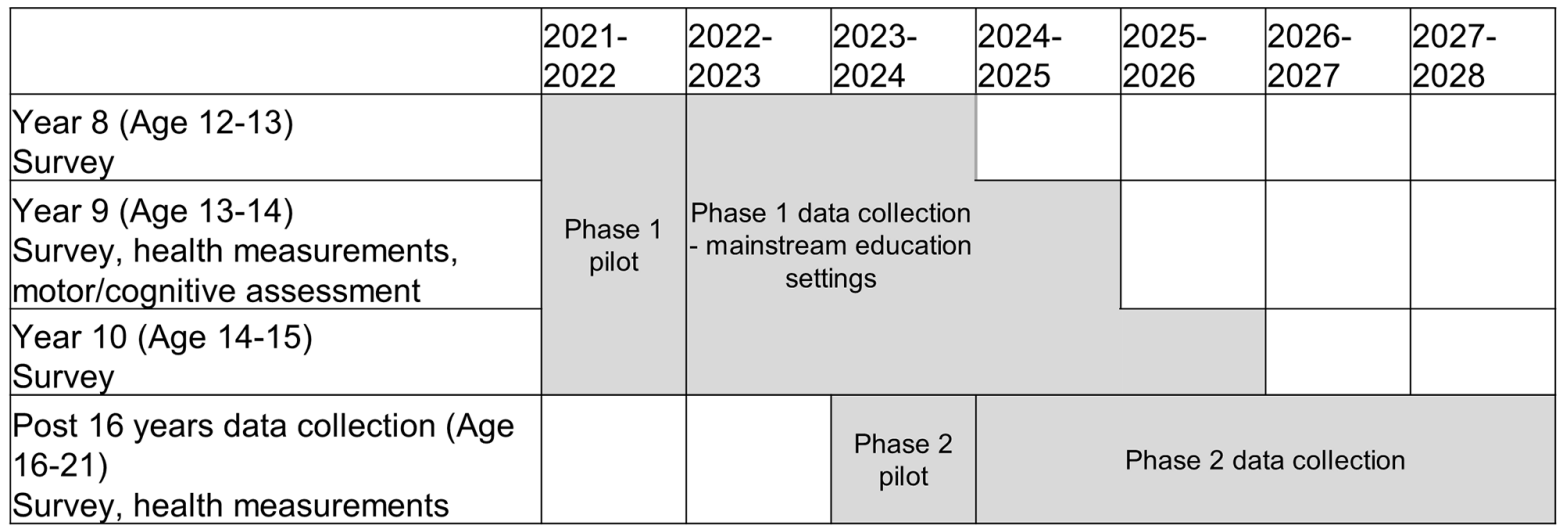
Quantitative data collection points for young people through Born in Bradford (BiB) Age of Wonder. Type of data collected for each age group is shown by calendar year, for pilot and main data collection. Phase 1 covers data collected in Years 8–10, whilst the young person is in secondary school. Phase 2 will cover data collected post-secondary school, when the young person is aged 16 years or older.

Within Work Package 2, there will be two phases of data collection of characteristics that will be analysed through quantitative methods, for example data on sociodemographic and behavioural characteristics captured in questionnaires, measurements of blood pressure and anthropometry and collection and storage of blood samples for biomarker assessment. In the first phase, young people aged 12–15 years old (school years 8–10) will be recruited into AoW through participating secondary schools. Phase 2 will follow the AoW participants from the age of 16 years, in schools, further education or community settings, until the age of 21 years old (see Figure 2).

### Scope of paper

This paper focuses on Work Package 2, the quantitative methods, in Phase 1 of data collection (young people aged 12–15, in mainstream secondary schools). This data collection began in 2022 and will continue until 2026. Methods were piloted with six schools between January and July 2022, with full data collection commencing in September 2022. Details of our longitudinal qualitative methods (Work-package 3) are published elsewhere ( Dogra *et al.*, 2023).

The co-production work from Work Package 1 that was carried out to design these methods is described further below, alongside the lessons learnt and adaptive changes made during the first year of data collection.

## Methods

### Setting

Bradford is the 7th largest metropolitan district in England with a population of > 540,000 ( City of Bradford Metropolitan District Council, 2022). It has a multi-ethnic population with the latest census data from 2021 showing that 61% of the population identified as White British, and 32% as Asian/Asian British (predominantly of Pakistani heritage). It is a socio-economically deprived city, with 34% of Bradford residents living in areas that rank in the most deprived decile of local areas in England ( Ministry of Housing Communities and Local Government, 2019), with high levels of health inequalities ( Public Health England, 2020).

### Co-production

Co-production is at the core of the AoW, and follows the established City Collaboratory approach ( Albert *et al.*, 2023; Wright *et al.*, 2019) which involves researchers, practitioners, decision makers and young people working together in equal partnership.

Since February 2021, AoW has held over 85 sessions with 12 separate groups, working with over 180 individuals, including young people, teachers, parents, and community members from varied ethnic backgrounds. Whilst co-production has involved a broad range of groups and individuals, co-production efforts have primarily involved five key groups: the Healthy Minds Apprentices, the BiB Young Ambassadors, the Bradford District Care Trust Young Dynamos, BiB Parent Governors (parents) and the AoW Teachers Group (teachers) (additional details provided in supplementary file 1, available as *Extended data* ( Shire, 2023)).

Co-production activities initially focused on priority setting, methods and development of study materials. As the project matures we will make use of our key forums to aid with dissemination and impact. Groups worked collaboratively with the research team to prioritise topic areas, and ensure the feasibility and acceptability of methods. In the development of survey measurements, young people scrutinised content, and suggested changes, additions and omissions. Where concerns were raised about validated measures which were not able to be amended, the groups suggested additional explanatory text to ensure understanding of why the questions were being asked. The groups also helped to design the methods for the anthropometrics and blood sampling. For example, in relation to concerns about weight stigma, our protocols ensure that measurements are conducted in private spaces, and no readouts can be seen by the students themselves.

A suite of opportunities have been developed for the young people involved in the co-production groups, enabling them to upskill themselves and gain experience; subsequently young people have presented at collaborator meetings, conferences, festivals, undertaken work experience and placements on the project, and pitched and developed content for our website and social media platforms.

### Sample size and power considerations

Up to 30,000 young people will be recruited; of these we estimate that around 10,000 will be existing BiB participants. Potential power for a range of example exposure and outcome relationships can be found in supplementary file 2 ( Shire, 2023).

### Measurements

***Questionnaires.*** The content of the questionnaires was co-produced with young people, teachers, and academics. A range of validated measures were included for direct comparison with other large scale adolescent studies in addition to bespoke measures that were suggested by external stakeholders (e.g., young people, Public Health, Bradford City of Culture 2025, District Nurse Team). The questionnaire data were collected and managed using REDCap (RRID:SCR_003445) electronic data capture tools hosted at Bradford Teaching Hospitals NHS Foundation Trust ( Harris *et al.*, 2009; Harris *et al.*, 2019), including the REDCap Mobile app ( Harris *et al.*, 2021). REDCap (Research Electronic Data Capture) is a secure, web-based software platform designed to support data capture for research studies, providing 1) an intuitive interface for validated data capture; 2) audit trails for tracking data manipulation and export procedures; 3) automated export procedures for seamless data downloads to common statistical packages; and 4) procedures for data integration and interoperability with external sources.

The key topics can be seen in Table 1, and the full question bank for the school year 2022–2023 can be seen in supplementary file 3 ( Shire, 2023).

***Motor and cognitive measurements.*** There are a variety of different tasks that comprise the full battery (see supplementary file 4 for full list of tasks ( Shire, 2023)) that can be broadly construed as either motor or cognitive. These tasks were previously used in the primary school sweep of data collection for the BiB cohort ( Hill *et al.*, 2021). The motor tasks are designed to assess aspects of hand-eye coordination which may function as barriers to success in the classroom and/or be associated with certain neurodevelopmental disorders. Similarly, the cognitive assessments have been selected as indicators of academic success and/or behavioural difficulties. In addition to the original measures, we also expanded the battery to incorporate a new language measure.

**Table 1.** Overview of questionnaire topics and key validated measures for Age of Wonder questionnaires 2022-2023.

Module	Topic	Validated Measures
Module 1	Socioeconomics	Family Affluence Scale (FAS) ( Currie *et al.*, 2010) Striving Against Inferiority Scale (SAIS) ( Gilbert *et al.*, 2007)
	Arts and Culture	
Module 2	Mental Health and Wellbeing	Depression/Anxiety (RCADS-25) ( Ebesutani *et al.*, 2012) Wellbeing (SWEMWBS) ( Shah *et al.*, 2018) Loneliness (UCLA loneliness scale 4) ( Russell, 1996) General Help Seeking (GHSQ) ( Wilson *et al.*, 2005) Resilience (BRS) ( Smith *et al.*, 2008) Psychosis (PLIKS-8) – Year 10 only ( Zammit *et al.*, 2013) Perceived social support (MSPSS) ( Zimet *et al.*, 1988)
	The Environment	
Module 3	Physical Activity	Eating disorders (EDE-QS) ( Gideon *et al.*, 2016) Physical Activity (PAQ-A) ( Kowalski *et al.*, 2004) Sedentary Activity (YAP: Sedentary) ( Saint-Maurice & Welk, 2015)
	Risk Behaviours	
Module 4	School Digital and Social Media	Pupils Attitudes Towards Technology (PATT) ( Ardies *et al.*, 2013)
	Identity and Discrimination	Adolescent Discrimination Distress Index (ADDI) ( Fisher *et al.*, 2000)

The tasks are run on Microsoft Surface Pro 8s with Wacomm BAMBOO ink-plus styluses.

***Health measurements.*** Height, weight, body composition via bioimpedance and skinfold thickness, blood pressure and a blood sample are collected (see supplementary file 5 ( Shire, 2023)) for full list of equipment used.

### Recruitment

***Schools.*** Recruitment targeted 37 of the largest mainstream secondary schools in the Bradford District. These schools were selected as they contain large amounts of existing BiB participants. Recruitment strategies include contacting headteachers and Chief Executive Officers (CEOs) of multiple academy trusts directly, utilising contacts through other schools, existing groups within the education system (such as the Education Alliance for Life Chances) and the local authority, identifying and attending local headteacher briefings, and members of the team representing BiB at over 50 events including careers fairs, webinars, and local authority events. Schools are offered £1000 per year for participation as a contribution to the costs of participating. We also work with schools to develop bespoke opportunities to develop workshops on research skills, data science (including data handling and interpretation), life-course approaches to health, and careers.

Once a school agrees to participate in AoW, the headteacher is asked to sign a consent form and data sharing agreements on behalf of their school.

***Young people.*** For the young people involved in this phase of data collection, we ask for parental consent, and participant assent.

There are two consent processes for our measures, an opt-out process for the less intrusive measures (questionnaires, motor and cognitive measures and most health measures), and an opt-in process for the blood sample.

Parents/carers are informed about their child’s data being used for research in BiB and being made available anonymously to other researchers worldwide. They are also informed that their child's data may be used to evaluate interventions that may be introduced in school.

Inclusion criteria:

- Young person attending a school in the Bradford Local Authority district that has consented to take part in BiB AoW
- In school years 8 (age 12–13 years), 9 (age 13–14 years) or 10 (age 14–15 years)
- Research team able to contact parent / carer of young person to provide them with information about the study

Exclusion criteria:

- Parent/carer does not consent
- Young person does not provide assent.
- Teacher decides it is not appropriate for young person to take part

Parental information sheets and consent forms can be viewed here. Video versions are available in English and Urdu, and each school is involved on a bespoke basis to ensure clear communication for their parents/carers. This can involve additional activities such as attending parents evenings or liaising with school parent involvement worker.

We share information about the study with the young people at school assemblies and at the time of each of the measurements. We ask the young person’s assent to take part in each measure. For the questionnaires, they have full autonomy over answering any or all questions.

### Procedure

We work with schools’ nominated lead to plan the most convenient dates and times for the measurements. AoW is launched at each school with an assembly with each of the year groups. The purpose of the research is explained, along with what will happen for each measurement, the processes in place to ensure privacy, and how their data is kept safe. Opt-out consent forms and information sheets are distributed to pupils to give to their parents/carers. These documents can also be sent electronically through schools’ regular communication channels with parents.

Once the school is satisfied parents/carers have been informed and have had a chance to consider the information and opt-out (normally over a two-week period), they are then asked to share a class list with the AoW team of those pupils who have not been opted-out. This class list includes demographic information listed on their school records (e.g. name, date of birth), as well as school email address.

### Measurements with opt-out consent

***Questionnaires.*** Questionnaires can be administered online by school staff by sending a unique link to the student’s email address, or offline using tablet computers supervised by members of the research team.

To reduce participant burden the questionnaire is split into four modules in the pilot and first year of data collection. These are delivered either separately in four 20–30-minute sessions, or back-to-back across two 50-minute sessions.

Prior to each session, a short presentation or video introduction is given to the topics covered. A list of resources (e.g. Youth in Mind mental health services) for students to access if any of the topics affect them is also provided.

***Motor and cognitive measurements.*** The assessments are administered in a single 50-minute session in a whole class setting. Up to 30 students and three members of research staff are present in each session. The session begins with a brief introduction to the “brain games”, with participants then working through the games simultaneously, with verbal instructions being delivered to the group before each of the tasks.

***Health measures.*** These measures are collected in the school during a bespoke clinic. Participant visit sheets are generated prior to attendance at school, showing the participant’s personal details with a unique barcode for tracking their data.

Private spaces are preferred where available (e.g., school health and well-being centre). Where this is not possible, school halls or classrooms are utilised, with screens separating students who are receiving assessments to ensure privacy.

At each assessment station, the participant’s personal details are checked, their visit sheet barcode is scanned into the assessment device, verbal assent is obtained, the assessment is completed, and completion of the assessment is logged on the participant’s visit sheet. The barcode pseudonymously tracks the participant’s data in the study servers once synchronized. If the young person takes part in all the measures, the assessment process lasts approximately 20 minutes.

### Measurement with opt-in consent

***Blood sample.*** At a separate school assembly (and after the opt-out process has been completed), Year 9 students receive a bespoke presentation explaining the blood sample process, and the importance of this data collection. The young people are given opt-in consent forms and an information sheet to take home to their parents/carers (see here), and this is also sent electronically by the schools via their regular communication channels with parents, with an option to consent online. Parents/carers of those who are part of the original BiB cohort have already consented to the linkage of their contact information. As such, the research team will call these parents to further encourage them to complete the consent form and answer any questions. On the day of the visit, the same process is followed as with the health measures.

### Feedback of results

Several data dashboards have been built to provide interactive feedback, and which will be updated at the end of each academic year. For example, the *Age of Wonder Data Dashboard for Senior Leadership* provides aggregated data for students at participating schools, permitting senior leadership to report and act upon any areas of concern. The prototype was initially built in consultation with previous studies that had provided dashboards for schools ( BeeWell and Oxwell). Senior leadership from participating schools were subsequently invited to a demonstration and given the opportunity to provide their thoughts and suggestions for the final co-design. All prototyping was done in RShiny (RRID:SCR_001626; Chang *et al.*, 2022) with the final product ported over to Knowage, a free, open-source platform that enables secure data reporting over the web.

Schools are able to compare categorical and continuous outcome measures against other participating schools across the city, and with age-matched reference samples where available. Moreover, schools are able to filter and group data on the primary characteristics they use for all their routine internal reporting (year group, sex, ethnicity, free school meals, and SEND status). Any groups that fall below a conservative threshold (n <16) will not be displayed to preserve anonymity and prevent spurious findings. Signposting for further information and support is also given to make this data actionable. The dashboard was launched with an instructional webinar to ensure responsible use.

An *Age of Wonder Data Dashboard for Students* has also been developed. This is delivered on request in the context of interactive data science workshops by a member of the research team. Students learn about basic statistical and visualisation concepts, exploring the topics of interest to them through their school’s aggregated results compared to the rest of the cohort.

In conjunction with GP Alliances and Community Pharmacy colleagues, a clear standardised procedure and pathway to feedback abnormal blood sample results and vitamin D levels to parents and GPs was developed. A similar pathway for blood pressure results is currently being developed.

### Data analysis plan

Research questions will be tested initially using multivariable regression analyses. Multivariable linear regression (with transformation/categorisation of variables, and exploration of non-linear relationships as appropriate) will be used for continuously measured outcomes and multivariable logistic or multinomial regression for binary/categorical outcomes. Where outcomes are repeatedly assessed (e.g. mental health, growth) multilevel models will be used (with measurements clustered within individuals) both to explore any change in associations with these outcomes as young people age, and to deal with missing data (under a missing at random assumption these methods allow all participants with at least one measure to be included).

### Ethical considerations

The methods described in this paper were reviewed and approved by the Bradford Leeds NHS Research Ethics Committee [Ref: 21/YH/0261, date: 22.12.21]. The key ethical considerations for this project are:

***Participant burden.*** The study will involve inviting young people to take part in repeated questionnaires, and for a subsample, additional measurements and a blood sample. Young people have told us very strongly that questionnaires should be as short as possible, and it should be clear to young people why we are asking questions. In order to minimize burden we have worked with schools to implement a modular approach to data collection, chunking questionnaires into 15–20 minutes modules which can be accessed at convenient times during the school day. Measurements (e.g. height, weight, bio-sampling) will be done during the schools day. Young people (and parents) will be free to withdraw at any time.

***School burden.*** Implementation of AoW research activities means there is some disruption to class timetables and also a burden of time for school staff involved in helping to arrange logistics. A flexible approach is taken with schools to work around their preferences where possible, and schools are given £1000 each year in recognition of this administrative burden.

***Safeguarding,*** Young people will be asked to complete surveys on mental health and other sensitive topics in schools. As these will be collected at scale it is not possible to check questionnaires for responses that are indicative of very poor mental health in real time. The AoW team have worked very closely with young people to co-produce data collection activities to ensure that they are acceptable. Trigger warnings have been included in the questionnaires and young people are reassured that they do not need to answer any question that they do not wish to. A booklet is provided which includes links to support they can access if they are affected by any of the topics. This has been co-produced with young people and services in the district such as Youth in Mind mental health support services. Young people are also encouraged to speak to a member of school staff if they wish to discuss anything from the questionnaires that may be of concern to them.

Where researchers are in schools and working directly with the young people, for example during the health assessments, there is the potential for participants to make disclosures that require a safeguarding referral to be made. If a disclosure is made it is the responsibility of the staff member to seek advice from the Designated Safeguarding Lead or a member of the school Senior Leadership Team in accordance with the safeguarding policies in place at the school. Research staff complete all relevant safeguarding training before any school visits are made.

During the health measures visits there is also the potential for members of the research team to witness self-harm marks which denote a young person experiencing emotional distress. In this situation the research team member will escalate this to the research nurse or co-ordinator who will ensure the young person has support in place going forward through discussion with the Designated Safeguarding Lead or a member of the Senior Leadership Team.

***Collection of blood samples.*** Should a first attempt to take blood be unsuccessful, a second attempt will be undertaken. If the second attempt is unsuccessful no further attempts during that session will be made. A repeat appointment at a later date can be offered if acceptable to the young person. If a young person objects or becomes distressed at any stage of the blood sampling the procedure is stopped.

Students can experience vasovagal syncope symptoms (faints) which include a temporary drop in blood pressure, pallor, sweating, shaking and nausea. To reduce the chances of this, staff are trained to recognise and respond appropriately when this occurs. For venepuncture a pre and post session ‘Safety Huddles’ ( Aldawood *et al.*, 2020) is conducted.

There may be some cultural sensitivities to taking blood during the period of Ramadan. Feedback from parents has suggested that taking of blood may not be perceived as acceptable for children who are observing fasting throughout this period. Where this is an issue the researchers work with parents and schools to offer alternative appointments.

***Health measures.*** Co-production with young people and schools has identified weight as a sensitive issue. For blood pressure and skinfold measures, young people are also asked to remove their left arm from their clothing. To mitigate this, young people are reassured that all assessments take place in a private booth, and they are not shown the results of the measures. They are reminded they do not have to take part in any measure they feel uncomfortable with.

***Data Management.*** Personal information is kept securely in accordance with the General Data Protection Regulations (GDPR) and Data Protection Act (2018). Pseudo-anonymised IDs are used to link survey data with participant details. Data is cleaned, documented and prepared for analysis by in-house statisticians and data management specialists following established standard operating procedures. Data item naming is coordinated according to in-house conventions; SQL Server database security roles separate administrative from research data use. Only authorised project administrators and database managers with a need to know can access participant personal identifiers. All research data and documentation is catalogued, maintained and archived in security-managed folders on the hospital storage area network. This is scalable, secure and backed up nightly. Born in Bradford’s research analytic programme manager acts as Data Custodian.

***Study status.*** At the time of protocol submission, the study is actively recruiting.

## Discussion

The AoW research programme aims to collect information on up to 30,000 young people as they navigate the sometimes turbulent journey from adolescence to young adulthood. School-based recruitment was chosen as this was found to be an efficient method of data collection. However, working with schools and young people in this way can be challenging. During the pilot phase (from January 2022 to September 2023) AoW worked with 15 schools over two academic years and experienced a range of unanticipated challenges. By sharing some lessons learned it is hoped that we can support other researchers who wish to work in this way.

### Lessons learned from pilot and co-production

Schools face a large number of challenges and competing demands on staff time. Additional burdens, such as changes in leadership, Ofsted inspections, cyber-attacks and staff sickness often severely impacted the capacity of the schools to engage with us. It was important to tailor our data collection activities to school preferences. We developed a detailed record of each individual school, including demographics, school priorities, key contacts, preferred methods of contact and notes from any visits by the research team on things like suitability of suggested spaces for testing. We added to this data capture form as we progressed through the project, allowing us to build up a tailored offer to each school, while maintaining the fidelity of the data collection itself.

During the first year of data collection, we found the four module questionnaire to be challenging; whilst we had good completion for the first two modules, there was a large drop off in completion of later modules due to being unable to schedule addition time in school. We therefore made the decision to reduce the length of the questionnaires by 50% for the year 2023–24, such that the survey is deliverable in a single 50-minute session or two separate 20–30-minute sessions. To identify the items to cut, we have used response rates/distributions of the data collected thus far, insight from the qualitative arm of the study, consultations with young people, and advice from our co-applicants, academic partners, and external stakeholders.

The availability of computers within the schools for students to complete the questionnaires was also a barrier to some schools agreeing to take part. Where this is a problem, we will visit the school with tablets and run data collection offline for groups of up to 100 young people.

Despite initial concerns from some schools, we have not experienced any concerns or reluctance from students around measuring weight. However, in two schools, there were students who declined the bioimpedance measure due to not wanting to take their socks off. Similarly, for the skinfold measurements, students have declined as taking part involves baring part of their torso for the subscapular measure. Some school uniforms mean students would be unable to do this easily. Aside from personal preference, the research team have also noted that in some cases, if one student declines, and there is then a knock-on effect where a whole group follow suit. To counter this we now check in one student at a time with any other students waiting a distance away.

Some of our activities require substantial amounts of heavy equipment to be transported from our research base into school settings. At times up to 100 tablets or equipment to construct multiple private booths needs to be transported. Training on safely handling equipment (etc) is given to team members. Furthermore, as sessions often started early in the school day, we aim to arrange equipment transport and set up booths the day before the data collection sessions to give team members the time to employ safe manual handling procedures.

In spite of the challenges described above, feedback from schools who took part in the study was overall extremely positive, with particular excitement around the data dashboards and how transformational these have the potential to be for school insights and planning. Given that in many cases, the schools that would benefit most from our data insights are the ones that have the least capacity to engage, it is our responsibility as researchers to make our ‘ask’ as easy as possible and demonstrate the benefits of the research in language that speaks to the school’s priorities.

### Planned dissemination

We have a number of strategies to further spread our learning to communities and students across Bradford. We are developing a suite of opportunities and training such as work experience, workshops, curriculum enhancement, social media training and leading panels. We run a regular Born in Bradford science festival for young people and members of the general public. Our inaugural event held in July 2023 saw over 200 pupils attend to lead and contribute to panels and workshops, and learn about the impact of the work they are contributing to. We are working closely with young people in schools, our co-production groups and through Bradford Citizens to upskill young people in using our data and the young people’s data dashboard. Alongside this, we are disseminating our work through our TikTok and Instagram social media channels, BBC radio 4 podcasts ( Robinson, 2023), newspaper articles and podcasts and workshops designed or led by the young people themselves.

We aim to establish BiB AoW as a positive force for change in the city, creating a sense of ownership from our young people, the schools and local policy makers and service providers. The methodology we have described here for phase 1 of the quantitative data collection is centred around embedding these measures in existing systems and ensuring that young people’s voices are recognised and amplified throughout the research programme.

The pace of research can be slow and this can be a source of frustration to policy and decision makers who need data to inform service provision. AoW has been developed in response to stakeholder priorities and we have a commitment to sharing data and findings ‘in real time’. Our datasets are designed to be shared at the end of each academic year, and our data dashboards will enable teachers and public health professionals to interrogate anonymised datasets to find areas of health and educational need. We are working closely with the team in the Local Authority Public Health team, who have input into the questionnaires, allowing frequent opportunities to inform policy making and influence service delivery.

## Data Availability

No data are associated with this article. Mendeley Data: Born in Bradford’s Age of Wonder cohort: protocol for adolescent data collection - extended data. https://doi.org/10.17632/xfgc4zjw62.1 ( Shire, 2023). This project contains the following extended data: Supplementary File 1 Co-production table Supplementary File 2 Power Calculations Supplementary File 3 Question bank for Age of Wonder Surveys 2022–23 Supplementary File 4 Table of Motor and Cognitive Tasks Supplementary File 5 Health Measurements and Blood Sample Procedure Data are available under the terms of the Creative Commons Attribution 4.0 International license (CC-BY 4.0).
