# Supplementary material for: Born in Bradford’s Age of Wonder cohort: protocol for adolescent data collection
Source: Wellcome Open Res. 2024 Feb 12;9:32. [Version 1] doi: 10.12688/wellcomeopenres.20785.1 (PMC11103777; doi:10.12688/wellcomeopenres.20785.1)
Supplement: Peer review history

# Reviewer report: Reviewer response for version 1

**DOI:** 10.21956/wellcomeopenres.23002.r76767

**Published:** 2024-05-17

**Authors:** Mia Eisenstadt

**Affiliations:** 1 Evidence Based Practice Unit, A partnership between UCL and the Anna Freud Centre, London, England, UK

Dear authors, 

 This is a fascinating protocol for an important quantitative study of the BIB Age of Wonder cohort that will capture data from multi-ethnic adolescents on a range of physical health and mental health outcomes, using a methodology that has involved considerable co-production and lessons learned from the first year of data collection.

 Overall, the protocol is thorough, and well constructed. It conveys attention to detail and resourcefulness and consideration in overcoming obstacles to data collection. The research project is ambitious and appears to have the potential to serve the communities studied within the research cohorts. 

 I do think the rationale for the study could be clearer. For example, we learn about the interest in obesity from the key words and not from the Abstract. Could the text be expanded to emphasize why this study is needed and who could benefit?   

**Suggested changes**

 -In the abstract can you be clearer about the specific outcomes that you are looking at. 

 -In the introduction you do not mention what outcomes you are interested in and why, instead the text goes straight into the methodology of taking cord blood samples. Could a sentence or two be added about why this cohort is at an elevated risk relative to other geographies and what are the benefits of conducting such a study, how will it help specific communities in Bradford in the future? 

 -Would suggest not scheduling any blood sampling for Ramadan which ended 10th of April and so next year will be Feb-March rather than giving children the opportunity to opt out. 

 -Is there something that the students can receive for participating in the study such as a certificate for participating in the research? 

 -Can you make it clearer for how long the data will be stored?

 -Health measures: is it possible for participants to request a same sex researcher when asked to remove the left arm of their clothing? This might be particularly relevant for female participants. 

 This text could be clearer: 

 "We aim to establish BiB AoW as a positive force for change in the city, creating a sense of ownership from our young people, the schools and local policy makers and service providers."- Is this a force for change towards greater mental health, cardiovascular health and reduced risk of obesity, or reducing health inequalities or something else- please specify. 

 -I notice that you have a measure that covers discrimination. Would this cover racism and intergenerational trauma? 

 - Might be worth considering a "life course approach" where racism and discrimination can be experienced across the life course.

Is the study design appropriate for the research question?

Yes

Is the rationale for, and objectives of, the study clearly described?

Partly

Are sufficient details of the methods provided to allow replication by others?

Yes

Are the datasets clearly presented in a useable and accessible format?

Not applicable

Reviewer Expertise:

Longitudinal research, adolescent mental health, youth participation in research.

I confirm that I have read this submission and believe that I have an appropriate level of expertise to confirm that it is of an acceptable scientific standard, however I have significant reservations, as outlined above.

## References

[1] : Measuring Structural Racism: A Guide for Epidemiologists and Other Health Researchers. *Am J Epidemiol* .2022;191(4) : 10.1093/aje/kwab239 539-547 10.1093/aje/kwab239 34564723 PMC9077112

# Reviewer report: Reviewer response for version 1

**DOI:** 10.21956/wellcomeopenres.23002.r74758

**Published:** 2024-05-07

**Authors:** Sonia Anand

**Affiliations:** 1 McMaster University, Hamilton, Ontario, Canada

**Study Protocol Born in Bradford Age of Wonder**

- Is the rationale for, and objectives of, the study clearly described?
  -  Yes, in summary they are extending and expanding the Born in Bradford (BiB) cohort study into adolescence using co-design methods with adolescence to develop interventions to improve health and well-being
- Is the study design appropriate for the research question?
   
  - Quantitative surveys of BinB participants and their peers in schools described as a large, whole city cohort that will capture the contemporary lived experience amongst multi-ethnic adolescents progressing into young adulthood. Adolescence is a high risk period for the onset of mental ill-health. They aim to collect repeated data from existing BiB participants and their peers (N~30,000 adolescents over 7 years).
  - “ *Young people aged 12-15 are being asked to complete questionnaires, covering topics such as mental and physical health. Those in Year 9 (13-14 years old), are also asked to take part in physical health measurements such as height, weight, blood pressure and a blood sample, as well as computer-based assessments of cognition (memory), movement and language.”*
  - These questionnaires have been co-designed with adolescents input.
- Are sufficient details of the methods provided to allow replication by others?
   
  - While and overview of the ambitious AoW program is presented, the detailed methods here are for Work Package 2.
  - Questionnaires can be accessed by following hyperlinks to https://data.mendeley.com/datasets/xfgc4zjw62/1
  - Power calculations are provided, a bit clunky to review in Mendeley presentation but all data there across maternal exposures and maternal and adolescence outcomes.
- Are the datasets clearly presented in a useable and accessible format:
   
  - This is described in the protocol, with summary data being shared on a dashboard at the end of each academic year and available to school and public health planners.
  - Their social media plans for dissemination of health learning is also well outlined.

**Overall Assessment:** Approved for indexing.

**Comments:**

- Please add more details regarding conversation with REB for the Opt out consent and Opt in for blood – was that difficult to get consensus on from REB and from Schools?
- Beyond the DASHBOARD and future publications, how will outside parties be able to apply to use the AoW data?
- More details on processing of blood and storage, and specific consent regarding DNA

Is the study design appropriate for the research question?

Yes

Is the rationale for, and objectives of, the study clearly described?

Yes

Are sufficient details of the methods provided to allow replication by others?

Partly

Are the datasets clearly presented in a useable and accessible format?

Yes

Reviewer Expertise:

My area of research includes but not limited to: birth cohorts, cardiovascular disease, vascular medicine, large randomized studies, intervention research, genetics, nutrition, qualitative research etc.

I confirm that I have read this submission and believe that I have an appropriate level of expertise to confirm that it is of an acceptable scientific standard.

# Reviewer report: Reviewer response for version 1

**DOI:** 10.21956/wellcomeopenres.23002.r74756

**Published:** 2024-03-05

**Authors:** Mary Barker

**Affiliations:** 1 University of Southampton, Southampton, UK

Overall:

 Thank you for sending me this article to review. I enjoyed reading it. It is very nicely written and clearly presented. It sets out the protocol for the first stage of the new Age of Wonder cohort study, a wonderfully ambitious endeavour that aims to recruit 30,000 young people between 11 and 15 years with follow-up over the subsequent seven years. The study involves a continuous process of co-creation of the research with young people and other stakeholders, quantitative and qualitative data collection, embedded intervention studies, translation of findings into impact at a local and national level and gives back substantially to the schools and young people who take part. The paper provides a description of the overall study and a protocol for the first phase which involves a quantitative survey of physical and mental health. The paper is excellent and I have very few comments.

 One thing that struck me is that it might be helpful to your readers for them to understand why the BiB cohort is being expanded by the inclusion of non-BiB young people in the Age of Wonder study. I wouldn’t have said that ‘to enhance the BiB resource’ is a sufficiently detailed or methodological justification for doing this.  Please could the authors add a little more explanation.

 It may also be worth the authors mentioning that they are aware of the groups of young people they might miss by recruiting only in a school setting.  

 I was slightly confused by the fact that the programme of data collection for the phase of research described in the protocol paper is designed in four modules.  In the discussion, it says that following piloting, the decision was taken to reduce the number of items and form only two modules for data collection.  What is the final protocol therefore? If it is for two modules then this should be presented as the final study design, not the four module version.

 These are all, however, minor comments which the authors can choose to take note of or not.  As I say, it is an excellent paper describing a brilliant piece of work and I have learned a great deal from doing this review. Thank you.

Is the study design appropriate for the research question?

Yes

Is the rationale for, and objectives of, the study clearly described?

Yes

Are sufficient details of the methods provided to allow replication by others?

Yes

Are the datasets clearly presented in a useable and accessible format?

Yes

Reviewer Expertise:

I am an academic psychologist working in public health, specialising in working with young people to improve their health and well-being.

I confirm that I have read this submission and believe that I have an appropriate level of expertise to confirm that it is of an acceptable scientific standard.
